# Evaluation of ERIC-PCR as Genotyping Method for *Corynebacterium pseudotuberculosis* Isolates

**DOI:** 10.1371/journal.pone.0098758

**Published:** 2014-06-05

**Authors:** Elaine M. S. Dorneles, Jordana A. Santana, Dayana Ribeiro, Fernanda Alves Dorella, Alessandro S. Guimarães, Mohamed S. Moawad, Salah A. Selim, Ana Luiza M. Garaldi, Anderson Miyoshi, Márcio G. Ribeiro, Aurora M. G. Gouveia, Vasco Azevedo, Marcos B. Heinemann, Andrey P. Lage

**Affiliations:** 1 Departamento de Medicina Veterinária Preventiva, Escola de Veterinária, Universidade Federal de Minas Gerais, Belo Horizonte, Minas Gerais, Brazil; 2 Departamento de Biologia Geral, Instituto de Ciências Biológicas, Universidade Federal de Minas Gerais, Belo Horizonte, Minas Gerais, Brazil; 3 Embrapa Gado de Leite, Empresa Brasileira de Pesquisa Agropecuária, Juiz de Fora, Brazil; 4 Departmento de Medicina Veterinária, Universidade Federal de Lavras, Lavras, Minas Gerais, Brazil; 5 Department of Toxicology and Forensic Medicine, Cairo University, Cairo, Egypt; 6 Centro Biomédico, Faculdade de Ciências Médicas, Universidade Estadual do Rio de Janeiro, Rio de Janeiro, Brazil; 7 Departamento de Higiene Veterinária e Saúde Pública, Faculdade de Medicina Veterinária e Zootecnia Universidade, Estadual Paulista, Botucatu, Brazil; Universidad Nacional de La Plata., Argentina

## Abstract

The aim of this study was to evaluate the Enterobacterial Repetitive Intergenic Consensus (ERIC-PCR) as a tool for molecular typing of *C. pseudotuberculosis* isolates from eight different hosts in twelve countries. Ninety-nine *C. pseudotuberculosis* field strains, one type strain (ATCC 19410^T^) and one vaccine strain (1002) were fingerprinted using the ERIC-1R and ERIC-2 primers, and the ERIC-1R+ERIC-2 primer pair. Twenty-nine different genotypes were generated by ERIC 1-PCR, 28 by ERIC 2-PCR and 35 by ERIC 1+2-PCR. The discriminatory index calculated for ERIC 1, ERIC 2, and ERIC 1+2-PCR was 0.89, 0.86, and 0.92, respectively. Epidemiological concordance was established for all ERIC-PCR assays. ERIC 1+2-PCR was defined as the best method based on suitability of the amplification patterns and discriminatory index. Minimal spanning tree for ERIC 1+2-PCR revealed three major clonal complexes and clustering around nitrate-positive (biovar Equi) and nitrate-negative (biovar Ovis) strains. Therefore, ERIC 1+2-PCR proved to be the best technique evaluated in this study for genotyping *C. pseudotuberculosis* strains, due to its usefulness for molecular epidemiology investigations.

## Introduction

*Corynebacterium pseudotuberculosis* is a Gram-positive, facultative intracellular bacterium, classified into two biotypes based on host preferences and nitrate-reducing activity. It is the causative agent of several infectious and contagious chronic diseases, including caseous lymphadenitis (CLA), ulcerative lymphangitis, mastitis, and oedematous skin disease (OSD), in a broad spectrum of hosts [1]–[5]. It is a common pathogen of sheep, goat and horses. It also causes sporadic infections in other species including cattle, buffalo, camels, llamas and humans [1]–[9].

In sheep and goats, *C. pseudotuberculosis* is etiological agent of the caseous lymphadenitis, predominantly caused by the nitrate-negative biovar Ovis strains [5]. This disease is widely distributed, with high prevalences in Australia [10], Brazil [11]–[13] and Argentina [14], where it is responsible for significant economic losses in wool, milk and meat production. In horses and water buffalos, *C. pseudotuberculosis* infection is responsible for ulcerative lymphangitis or chronic abscesses and edematous skin disease, respectively, being in both cases mainly caused by the nitrate-positive biovar Equi strains [3], [4]. Whereas, in cattle, *C. pseudotuberculosis* infection can be caused by both biovars and produces three clinical forms, cutaneous, mastitic and visceral, being the two last less common [1], [15], [16].

A great variety of DNA-based methods have been used for determining genotypes in individual isolates of *C. pseudotuberculosis*, including enzyme restriction of chromosomal DNA [17], [18], ribotyping [18]–[20], polymerase chain reaction - restriction fragment length polymorphism (PCR - RFLP) [21], Pulse-Field Gel Eletrophoresis (PFGE) [22] and Random Amplified Polymorphic DNA (RAPD) [23]. However, these techniques have revealed high genetic homogeneity within the species. This could reflect the clonal-like behavior of this pathogen or limitations in the methods used for strain identification.

Recently, our group proposed a typing method based on enterobacterial repetitive intergenic consensus-PCR (ERIC-PCR), which was shown to be a good test for genetic discrimination of *C. pseudotuberculosis* field strains from sheep and goats, with high resolution, repeatability and typeability [24], [25]. However, all previously typed isolates belonged to biovar Ovis and came from Brazil. Thus, the present study aimed to evaluate ERIC-PCR as a tool for molecular typing of *C. pseudotuberculosis* isolates from eight different hosts (buffalo, camel, cattle, goat, horse, human, llama and sheep) in twelve countries (Argentina, Australia, Belgium, Brazil, Chile, Egypt, England, France, Israel, Kenya, Scotland, and USA).

## Materials and Methods

### Bacterial Strains

Ninety-nine *C. pseudotuberculosis* field isolates, *C. pseudotuberculosis* ATCC 19410^T^ type strain and *C. pseudotuberculosis* 1002 vaccine strain were selected for genotyping by ERIC-PCR. These were representative strains selected from the collection of the Laboratório de Genética Celular e Molecular, Instituto de Ciências Biológicas, Universidade Federal de Minas Gerais, which receives strains from the most active laboratories on *C. pseudotuberculosis* research in the world. Moreover, they represent important *C. pseudotuberculosis* strains for the hosts or countries they were isolated in, as many of the bacteria used in the study have been selected for genome sequencing (CIP 52.97, PAT10, I19, FRC41, Cp31, Cp162, Cp267, Cp1002 and CpC231) [9], [26]–[32]. Information on host, country of origin and biovar of the *C. pseudotuberculosis* field isolates is summarized in the Table 1. This collection of strains includes several isolates from animals and a single isolate from a human, that were identified by routine phenotypical tests [5], and their species identification was confirmed by phospholipase D (PLD) PCR [33]. Among the 101 studied strains, 27 belong to biovar Equi and 74 to biovar Ovis (Table 1).

**Table 1:** Origin, hosts and biovars of 101 *C. pseudotuberculosis* isolates genotyped by ERIC-PCR.

Host	Biovar		Argentina	Australia	Belgium	Brazil	Chile	Egypt	England	France	Israel	Kenya	Scotland	USA	Total
	Equi	Ovis													
Buffalo	18	9	0	0	0	0	0	27	0	0	0	0	0	0	27
Camel	1	0	0	0	0	0	0	0	1	0	0	0	0	0	1
Cattle	1	3	0	0	0	0	0	0	0	0	4	0	0	0	4
Goat	0	30	0	0	0	29*	0	0	0	0	0	0	0	1	30
Horse	6	1	0	0	1	0	1	0	0	0	0	1	1	3	7
Human	0	1	0	0	0	0	0	0	0	1	0	0	0	0	1
Llama	0	1	0	0	0	0	0	0	0	0	0	0	0	1	1
Sheep	1	29	3	1	0	25	0	0	0	1†	0	0	0	0	30
Total	27	74	3	1	1	54	1	27	1	2	4	1	1	5	101

*One of the *C. pseudotuberculosis* strains is the vaccine strain 1002.

† *C. pseudotuberculosis* Type strain ATCC 19410^T^.

### ERIC-PCR

*C. pseudotuberculosis* genomic DNA was extracted as previously described [33]. The strains were fingerprinted by ERIC-PCR using the primers ERIC-1R (5′-ATGTAAGCTCCTGGGGATTCAC-3′), ERIC-2 (5′-AAGTAAGTGACTGGGGTGAGCG-3′) and the ERIC-1R+ERIC-2 primer pair (Invitrogen, Carlsbad, CA, USA) [34] as previously described [24]. Briefly, the PCR reaction was performed using 50.0 mM Tris, 1.5 mM MgCl2, 10.0 mM KCl, 50.0 mM (NH4)2SO4 (pH 8.3) (Phoneutria, Brazil); 0.2 mM of dNTP (each) (Life Technologies, USA); 2.0 mM of each primer (Life Technologies, USA); 2.5 units of Taq DNA polymerase (Phoneutria, Brazil) and 100.0 ng of template.

### Data Analysis

Band size estimates and genotype analyses were done using the software BioNumerics 6.5 (Applied Maths, Sint-Martens-Latem, Belgium). Clustering analysis was performed with the same software based on the Dice similarity coefficient and the unweighted pair group method with arithmetic mean (UPGMA). The Hunter and Gaston Diversity Index (HGDI) was calculated [35] for ERIC 1, ERIC 2, and ERIC 1+2. The typeability was evaluated from the proportion of isolates that were scored in the ERIC-PCR assays and assigned a type [36]. The three ERIC-PCR assays were also classified based on amplification pattern by evaluation of resolution of DNA amplification bands, average number of bands per genotype and presence of smearing.

The minimum-spanning trees (MST) were generated using the UPGMA to calculate the distance matrix Prim’s algorithm associated with the priority rule and the permutation resampling [37], [38]. The MST presented is the top score tree, the tree with the highest overall reliability score. Clonal complex term is frequently used in MLST (Multilocus Sequence Typing) analysis to describe patterns of evolutionary descent and is defined as a group of genotypes that share a minimum of 5 (a total of 7) (71.42%) *loci* [37]. Due to the great difference in resolution between the techniques (MLST *vs* ERIC-PCR), we determined, for ERIC-PCR analyses, to use clonal complex for a group of strains that share 100% of similarity on genotype analysis. However, for dendrogram analysis the clusters were classified based on ∼80% of similarity (ERIC 1 - labeled A–K; ERIC 2 - labeled A–I; ERIC 1+2 - labeled A–K).

### Statistical Analysis

The global agreement among the three techniques was calculated evaluating the number (n) of different genotypes in each assay of ERIC-PCR, per host or per country, using the nonparametric Kendall’s W statistic [39], [40] with the aid of R software version 2.9.0 (R Development Core Team, Vienna, WIE, Austria).

## Results

### ERIC-PCR Genotyping

**Figure 1:**
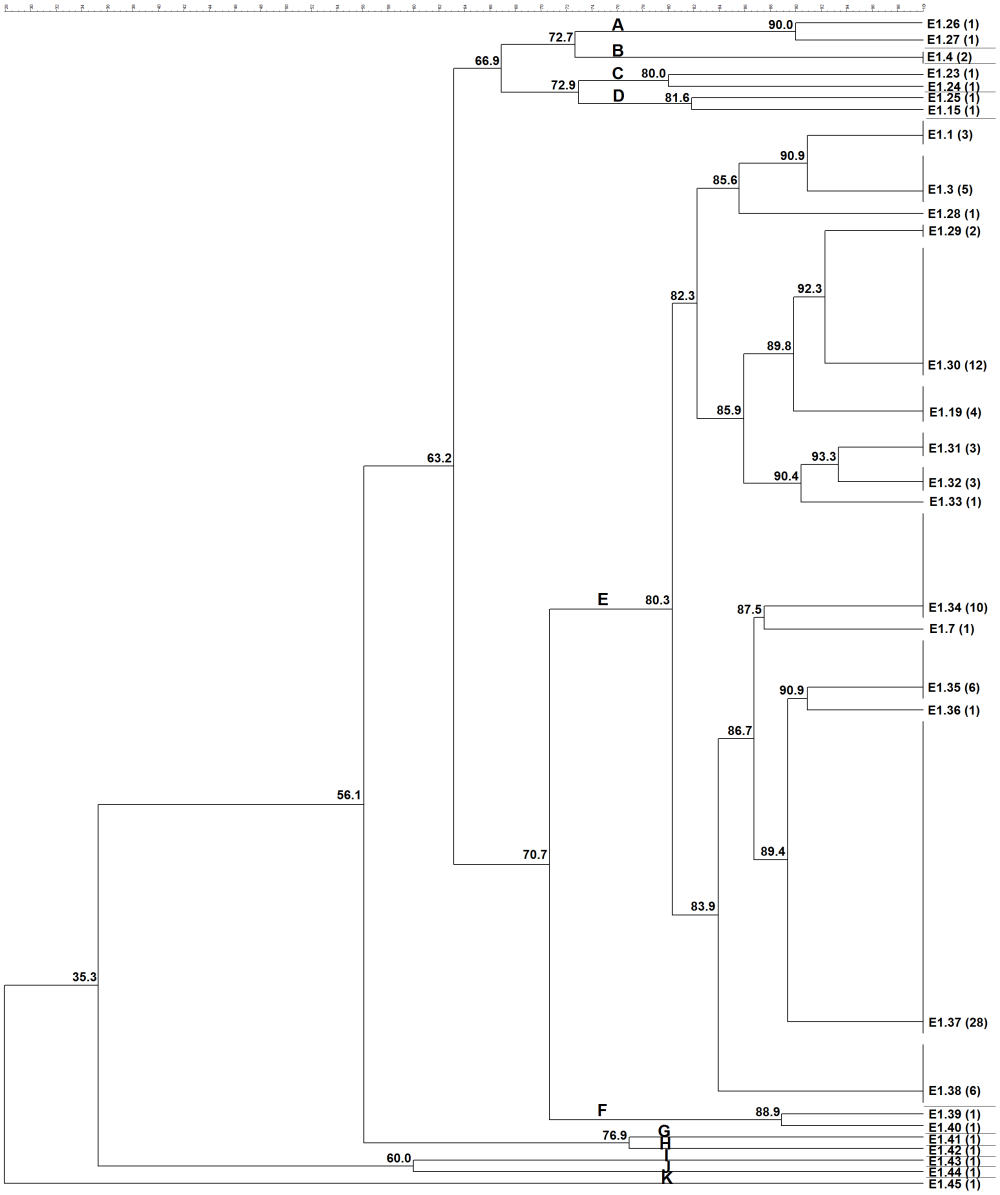
Cluster analysis by ERIC-PCR fingerprint (ERIC 1) of 99 *C. pseudotuberculosis* field isolates, 1002 vaccine strain and ATCC 19410^T^ type strain. Clustering analysis was performed with aid of BioNumerics 6.5 (Applied Maths, Sint-Martens-Latem, Belgium) and based on the Dice similarity coefficient and the unweighted pair group method with arithmetic mean (UPGMA). Eleven major clusters labeled A-K were defined from groups of closely related strains sharing on average ∼80% of genotype similarity. The numbers in parentheses correspond to the number of isolates within the genotype.

**Figure 2:**
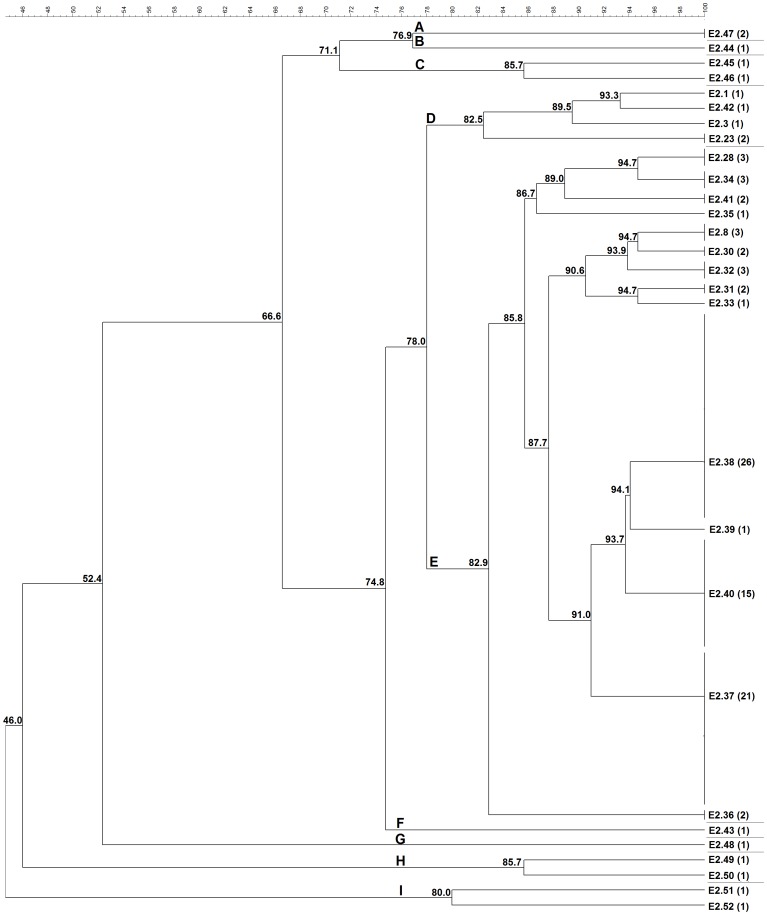
Cluster analysis by ERIC-PCR fingerprint (ERIC 2) of 99 *C. pseudotuberculosis* field isolates, 1002 vaccine strain and ATCC 19410^T^ type strain. Clustering analysis was performed with aid of BioNumerics 6.5 (Applied Maths, Sint-Martens-Latem, Belgium) and based on the Dice similarity coefficient and the unweighted pair group method with arithmetic mean (UPGMA). Nine major clusters labeled A-I were defined from groups of closely related strains sharing on average ∼80% of genotype similarity. The numbers in parentheses correspond to the number of isolates within the genotype.

**Figure 3:**
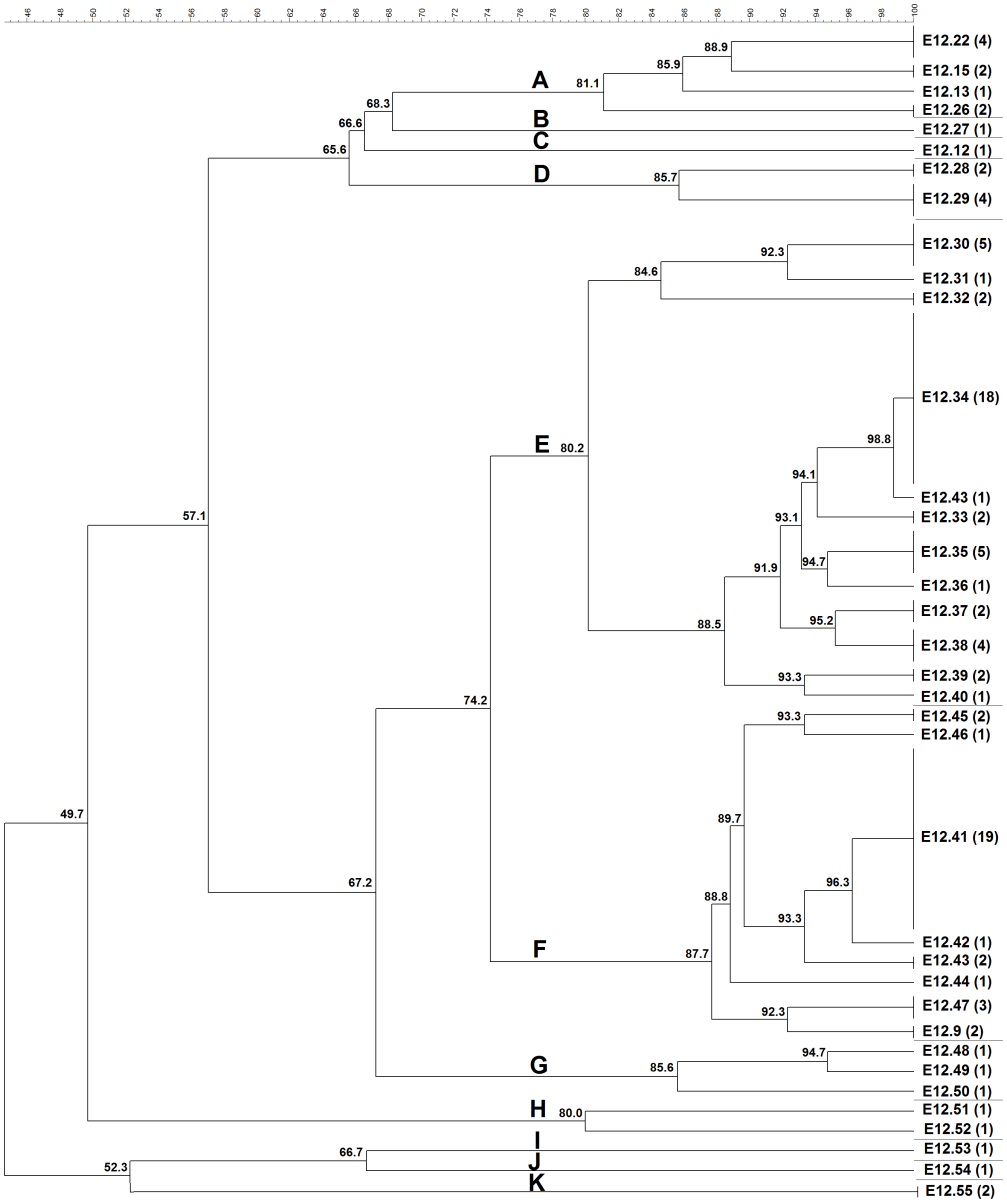
Cluster analysis by ERIC-PCR fingerprint (ERIC 1+2) of 99 *C. pseudotuberculosis* field isolates, 1002 vaccine strain and ATCC 19410^T^ type strain. Clustering analysis was performed with aid of BioNumerics 6.5 (Applied Maths, Sint-Martens-Latem, Belgium) and based on the Dice similarity coefficient and the unweighted pair group method with arithmetic mean (UPGMA). Eleven major clusters labeled A–K were defined from groups of closely related strains sharing on average ∼80% of genotype similarity. The numbers in parentheses correspond to the number of isolates within the genotype.

ERIC-PCR was able to fingerprint and assign a type to all the 101 *C. pseudotuberculosis* strains studied from different hosts and geographic origins. For all ERIC-PCR assays, the previously described genotypes were assigned the same identification label used by Guimarães et al. (2011) [24] and Dorneles et al. (2012) [25], and the new genotypes were labeled sequentially in the same way (Figures 1, 2, and 3).

Among all studied strains, ERIC 1-PCR resolved 29 genotypes (Figure 1), ERIC 2-PCR 28 genotypes (Figure 2), and ERIC 1+2-PCR 35 genotypes (Figure 3). The HGDI calculated for the ERIC 1-, ERIC 2- and ERIC 1+2-PCR were 0.89, 0.86, and 0.92, respectively. For ERIC 1-PCR, among the 29 genotypes resolved, 15.85% (6/29) had already been described for *C. pseudotuberculosis* strains isolates from sheep and goats of the Brazilian States of Minas Gerais and Pernambuco [24], [25], whereas the new genotypes constituted 84.15% (23/29) of all typed *C. pseudotuberculosis*. The E1.37 genotype was the most prevalent one containing 27.72% (28/101) of the tested strains, followed by E1.30 that occurred in 11.88% (12/101) of the strains. The average number of bands observed in genotyping by ERIC 1-PCR was 5.60, ranging ranged from 168.4 bp to 1,578 bp. Four bands were more frequent in 67.32% (68/101) of the strains in this assay. The molecular weights of the four frequent bands were approximately 168.4 bp, 342.4 bp, 589.2 bp and 652.7 bp.

Of the 28 genotypical profiles resolved by ERIC 2-PCR, five had been previously described [24], [25], whereas the genotypes of 90.09% (91/101) of all studied strains were new. The E2.38 genotype was the most prevalent one representing 25.74% (26/101) of the strains, followed by E2.37 with 20.79% (21/101) and E2.40 with 14.85% (15/101) of prevalence. Four bands were most frequent in 80.19% (81/101) of all strains. The molecular weights of the frequent four amplicons were approximately 195.1 bp, 378.8 bp, 430.84 and 666.5 bp, while all observed amplicons ranged from 195.1 bp to 1,290 bp.

Of the 35 genotypes determined by ERIC 1+2-PCR, only five had been previously reported [24], [25], whereas the genotypes of 90.09% (91/101) of all studies strains were not previously described. The most prevalent ERIC 1+2 genotypes were E12.41 and E12.34, which were observed in 18.81% (19/101) and 17.82 (18/101) of all tested strains, respectively. The average number of bands observed by genotypes in this assay was 7.35. The molecular weights of the frequent fragments were 104.6 bp, 391.8 bp, and 621.9 bp and they were found in 81.18% (82/101) of tested strains, while all PCR products by this reaction ranged from 94 bp to 1,282 bp.

### ERIC-PCR Clustering Analyses

In the ERIC 1-PCR dendrogram, cluster A was composed by one sheep strain from Argentina and another camel isolated from England, and cluster B consisted of one sheep and one goat strains from São Paulo State, Brazil. Cluster C was composed by one horse and one goat strains from Chile and Brazil, respectively, and cluster D by two USA horse isolates. The isolates grouped in cluster E (85.1%) originated from 27 sheep (23 from Brazil, two from Argentina, one from Australia and the ATCC 19410^T^ reference strain), 27 goats (26 from Brazil and one from USA), 26 buffalo (from Egypt), four cattle (from Israel), one horse (from USA) and one llama (from England). Each of clusters G, H, I, J and K were represented by a single isolate from buffalo (Egypt), sheep (Brazil), horse (Scotland), goat (Brazil) and human (France), respectively.

In ERIC 2 cluster analysis, cluster A was composed by one horse (Scotland) and one goat (Brazil) isolate, while cluster B was composed by a single human isolate, and cluster C of two isolates; one from buffalo (Egypt) and one from sheep (Brazil). Cluster D included four genotypes (five strains), originated from four goats (Brazil) and one sheep (ATCC 19410^T^ reference strain) strains. Cluster E comprised 84.1% of typed strains and was composed by 27 sheep (23 from Brazil, three from Argentina and one from Australia), 22 goats (21 from Brazil and one from USA), 24 buffalo (from Egypt), four cattle (from Israel), six horses (three from USA, one from Kenya, one from Belgium and one from Chile), one llama (from USA) and one camel (from England) strains. Clusters F and G included one sheep and one goat strain, both from Brazil, whereas clusters H and I were formed by two sheep (Brazil) and two buffalo (Egypt) strains, respectively.

In the ERIC 1+2 dendrogram, cluster A consisted of three sheep and three goats strains from Brazil, two buffalo isolates from Egypt, beyond the reference strain ATCC 19410^T^. Clusters B and C were composed by a single isolate, one goat (from Brazil) and one human (from France) isolate, respectively. Cluster D was formed only by sheep isolates, all from Brazil. Clusters E and F had most of the genotyped strains. Cluster E was composed by 11 sheep (eight from Brazil, two from Argentina and one from Australia), 22 goat (21 from Brazil and one from USA), seven buffalo (from Egypt), three cattle (from Israel) and one llama (from USA). Cluster F was composed by six sheep (five from Brazil and one from Argentina), six horse (three from USA, one from Belgium, one from Kenya and one from Chile), 17 buffalo (from Egypt), one cattle (from Israel) and one camel (from England). Cluster G was formed by two sheep and one goat strains both from Brazil. Finally, cluster H was composed by two Brazilian goat isolates, whereas cluster I, J and K included one buffalo (from Egypt), one sheep (from Brazil), and one horse (from Scotland) and one sheep (from Brazil), respectively.

The Kendall’s W coefficient of concordance observed among the three techniques of ERIC-PCR was 0.982 (P = 0.00438) when the results were grouped by host species and 0.991 (P = 0.00059) when they were grouped by country of origin. ERIC 1+2-PCR showed higher HGDI and better consistency, complexity and performance of DNA amplification than other assays tested.

### Clustering Patterns of Biovar Ovis and Equi Strains

**Figure 4:**
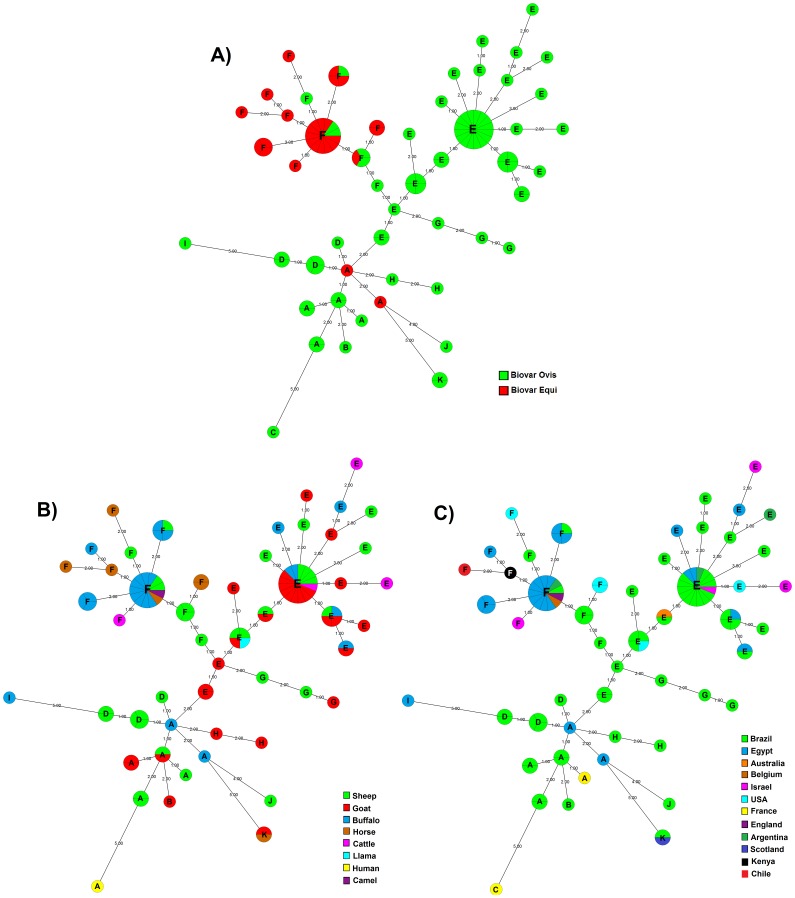
Minimal spanning trees (MSTs) by ERIC 1+2-PCR of 99 *C. pseudotuberculosis* field isolates, 1002 vaccine strain and ATCC 19410^T^ type strain. A) Clonal complexes according to biovar of the strains. B) Clonal complexes according to host origin of the strains. C) Clonal complexes according to country origin of the strains. The MST presented is the tree with the highest overall reliability score and were calculated using the UPGMA associated with the priority rule and the permutation resampling using Bionumerics 6.5 (Applied Maths, Sint-Martens-Latem, Belgium) [37], [38]. The number within of node indicates the cluster observed in dendrogram analyses (Figure 3). The numbers between two neighboring ERIC 1+2-PCR types indicate distance between them. The sizes of the nodes depend on the number of strains (their population size). Wedges in circles represent the proportion of *C. pseudotuberculosis* isolates from respective sources.

A MST was created based on ERIC 1+2-PCR fingerprint (Figure 4). The MST revealed the existence of three major clonal complexes that clustered around nitrate-negative (Ovis) and nitrate-positive (Equi) strains, although no specific genotypic profile was observed for *C. pseudotuberculosis* nitrate-positive and nitrate-negative strains by ERIC 1+2PCR. *Corynebacterium pseudotuberculosis* biovar Equi strains, with the exception of two strains isolated from buffalo, exhibited a pattern of clustering (cluster F), as well as observed for biovar Ovis strains, which were predominant in all other clusters but cluster F (Figure 4 panel A). All biovar Ovis strains grouped together with biovar Equi strains were sheep isolates, most from São Paulo State, Brazil, and one from Argentina.

## Discussion

*Corynebacterium pseudotuberculosis* has been considered an exceptionally homogeneous species, showing a low genetic diversity among isolates by several different molecular typing assays [17]–[23]. However, our recent results show that the amplification of DNA between successive repetitive intergenic consensus elements through ERIC-PCR is a good method for molecular characterization of *C. pseudotuberculosis* strains isolated from sheep and goat, with great discriminatory power and typeability, besides the good repeatability [24], [25]. In this study, our findings were broaden by characterizing with ERIC-PCR assays (ERIC 1, ERIC 2 and ERIC 1+2) a very diverse population of *C. pseudotuberculosis* isolates from both biovars, Ovis and Equi, including strains from eight different hosts (buffalo, camel, cattle, goat, horse, human, llama and sheep) isolated from twelve countries (Argentina, Australia, Belgium, Brazil, Chile, England, France, Egypt, Israel, Kenya, Scotland, USA) (Table 1).

Molecular typing methods are commonly used to investigate epidemiological relationships among isolates and sources of infection. However, before being used for those purposes, PCR methods for molecular typing require careful in-house validation of typeability, reproducibility, repeatability, stability, discriminatory power and epidemiologic concordance [36], [41]. Since Versalovic et al. (1991) [34] evaluated the ERIC-PCR technique for eubacteria, the method has been successfully applied for genotyping of different microbial pathogens, including gene mapping, detection of strain diversity, population analysis, epidemiology, and the demonstration of phylogenetic and taxonomic relationships [42]. Our data showed that, as reported in our previous studies with *C. pseudotuberculosis* isolates from Brazilian sheep and goat, the ERIC-PCR (all assays) showed high typeability, with all strains being fingerprinted and assigned a type. Moreover, the assays presented a high discriminatory power as shown by the high HGDI indexes observed. These high discriminatory power and typeability, associated with its high repeatability [24], supports the use of ERIC-PCR as a good molecular typing technique also for *C. pseudotuberculosis* strains.

Following the in-house ERIC-PCR validation, based on that neither laboratory nor epidemiologic evidence is definitive by itself, but each one validates the other [43], the epidemiological concordance of *C. pseudotuberculosis* genotyping by this method was established. Since, strains epidemiologically related (from same origin) exhibited identical genotypes (all ERIC-PCR), which in some cases were also identical to genotypes previously described for *C. pseudotuberuculosis* isolates from the same region [25]. Of the six epidemiologically related strains (331, 336, 445, 446, 447 and 453), for ERIC 1-PCR, four had genotypes identical to previously described ones, and for ERIC 2-PCR and ERIC 1+2-PCR the number of strains that had identical genotypes was four and five, respectively.

Moreover, of the types assigned by each ERIC-PCR, the majority (84.15% ERIC 1; 90.09% ERIC 2; 90.09% ERIC 1+2) correspond to new types not yet reported, which is consistent with the present sampling that is composed by only 6.06% (6/99) of field strains epidemiologically related with the previous samples [25]. The great number of novel genotypes observed may be the result of differences between hosts (sheep, goat, buffalo, horse, cattle, camel, llama and human), origin (Argentina, Australia, Belgium, Brazil, Chile, England, France, Egypt, Israel, Kenya, Scotland, USA), isolation year (1952 vs 2009) or evolutionary changes.

We also found that epidemiological concordance of the ERIC-PCR genotyping of *C. pseudotuberculosis* reflects epidemiological links observed in the formation of the sheep flock of Minas Gerais State, what corroborates our previous findings [24]. Data (2008/2009) from the state agency for animal health (Instituto Mineiro de Agropecuária - IMA) showed that there was a large transit of sheep from different states of Brazil (Distrito Federal, Espírito Santo, Goiás, Rio de Janeiro, Sergipe, São Paulo) into Minas Gerais, contributing to the constant growth of the state sheep population. Thus, validating the epidemiological data and vice-versa, some genotypic profiles found by Guimarães et al. (2011) [24] for *C. pseudotuberculosis* sheep isolates from Minas Gerais State were identical to genotypes observed for *C. pseudotuberculosis* sheep isolates from Pernambuco [ERIC 1 (E1.3), ERIC 2 (E2.1) and ERIC 1+2 (E12.22)] and São Paulo States [ERIC 1 (E1.1; E1.4) and ERIC 2 (E2.8)].

Regarding the three ERIC-PCR techniques used in this study, we had already previously shown that they were highly concordant among themselves, i.e., the genotypic differences observed by one of the techniques is very similar to differences observed by the others. This was also observed in the present study from a large sample of *C. pseudotuberculosis* isolates from different hosts and countries, which show high Kendall’s W coefficient of concordance. Thus, considering that the genetic diversity observed by ERIC-PCR assays are much alike, we selected two other parameters, HGDI and suitability of the amplification patterns, to define the best typing assay among the three ERIC-PCR assays for molecular characterization of *C. pseudotuberculosis* strains. The HGDI is a widely used index suitable to compare different typing systems [35]. DNA amplification results, which evaluates the consistency, complexity and performance of an amplification system, also allows the comparison among DNA-fingerprinting methods. Based on these parameters, we found that the ERIC 1+2-PCR is the best assay among the ERIC-PCR tested, since this assay presented the highest HGDI and a suitable amplification pattern providing more distinct DNA amplification bands, a good average number of bands per genotype and less smearing (data not shown). ERIC 2-PCR also demonstrated the same amplification characteristics of ERIC 1+2 primer set, however presented a low HGDI, despite having shown the best index in previous studies [24], [25]. In contrast, ERIC 1-PCR showed the lowest HGDI in all our studies [24], [25]. Furthermore, ERIC 1-PCR presented in all studies a less distinct and outnumbered band pattern. Versalovic et al (1991) [34] also observed that the primer ERIC-1 alone yielded limited amplification products. In addition, when the cluster analysis was based on similarity greater than 80%, classification by ERIC 1-PCR or ERIC 2-PCR depicted a large cluster composed by ∼85% of the strains.

One of the first techniques proposed to type *C. pseudotuberculosis* was biotyping, which divided the isolates in biovar Ovis and Equi, chiefly associated with strains isolated from sheep and horses, respectively [5]. Phenotypic characteristics can be linked to genotypes [42], as it was described for the nitrate-reducing ability related to different restriction patterns and ribotypes [17]–[19], whereas no genetic pattern between nitrate-positive and nitrate-negative *C. pseudotuberculosis* strains was depicted by ERIC-PCR. However, MST data analyses showed that, despite no association of nitrate reduction capability and ERIC-PCR genotypes, there was a clustering of isolates with similar results on nitrate-reduction test (Figure 4 panel A). Biovar Equi strains, with few exceptions, clearly clustered together (cluster F), while biovar Ovis strains were predominant in all other clusters but cluster F (Figure 4 panel A). Interestingly, all biovar Ovis strains grouped in cluster F were sheep isolates, most from São Paulo State, Brazil, and one from Argentina. The differences in the clustering pattern of biovar Ovis and Equi strains reflects the great number of genes not shared by both biovars, as complete genome analyses of 15 *C. pseudotuberculosis* strains showed that biovar Ovis contain 314 orthologous genes that are shared by all strain from this biovar but are absent from one or more strains of biovar Equi [44]. Furthermore, biovar Equi strains have 95 core genes that are absent from one or more strains of biovar Ovis [44].

The MST analysis also revealed the existence of three major clonal complexes, from which other clonally related isolated groups emerge, one in cluster F, the other in cluster E and another in cluster A (Figure 4). These inferences may become useful to develop a model for evolutionary steps in the difference of the *C. pseudotuberculosis* ERIC 1+2-PCR genotypes, nevertheless, more representative sampling is needed for inclusion into this model for a more robust comparison.

Associating the MST analysis with geographical or host origin is difficult because most of strains were isolated from a particular host belong to the same country (buffalo, goat and cattle), or because some strains are not well represented in the sample (camel, llama and man). However, the analyses of segregation of the strains with respect to geographical origin and host distribution among clusters determined by MST showed that, in spite of being the most heterogeneous geographical group (Table 1), all horse isolates were grouped in cluster F, as well others biovar Equi strains. One exception was a horse isolate from Scotland that was typed as biovar Ovis. This segregation pattern determined by biovar was also observed for water buffalo isolates, all from Egypt, which presented different clustering patterns: most of the biovar Equi isolates were in cluster F, whereas biovar Ovis isolates were grouped in clusters A, E and I, together with biovar Ovis isolates from other regions. Water buffalo isolates 45 and 49 were exceptions by being classified as biovar Equi and grouped in cluster A. Cattle strains also presented similar clustering patterns, with biovar Ovis strains being grouped into cluster E and biovar Equi strain grouped in cluster F. For buffaloes and cattle, which are not the main hosts of *C. pseudotuberculosis*, this biovar-clustering of isolates could be related to the host those species had acquired the infection from, since biovar Equi strains are closely associated to horse infection and biovar Ovis strains are mostly isolated from sheep or goat infection, representing, respectively, the usual cause of disease in horses and in sheep and goats [5]. Thus, it may also explain the clustering of camel, llama and human *C. pseudotuberculosis* isolates.

Sheep and goat *C. pseudotuberculosis* strains were grouped into several clusters and consequently were spread through different clonal complexes in MST. As most of the sheep and goat studied isolates (>90%) were from Brazil, those diverse types and clustering were probably due to the increase of the Brazilian sheep (14.8%) and goat (57.8%) commercial herds in recent years (1995 to 2006) [45], with intense traffic of animals sold for breeding and formation of new herds. Considering that the majority of sheep and goat isolates were from the Brazilian States of Bahia and São Paulo, respectively, it is noteworthy that São Paulo State had an increase of 86.1% in sheep herd population between 1995 and 2006, and that Bahia recorded a significant increase (11.3%) in its goat herd, the largest in the country, during the same period [45]. Furthermore, this growth in Brazilian sheep and goat herd population, due to an expansion of markets in all regions, was based in the importation of animals from various countries, mainly South Africa and Europe, which may have favored the entry and spread of different *C. pseudotuberculosis* strains and could also explain the genotypic similarity of isolates from Brazil with strains from those regions.

In conclusion, ERIC 1+2-PCR proved to be a good technique for genotyping of *C. pseudotuberculosis* strains, due to its usefulness for molecular epidemiology investigations.

## References

[1] Shpigel NY, Elad D, Yeruham I, Winkler M, Saran A (1993) An outbreak of *Corynebacterium pseudotuberculosis* infection in an Israeli dairy herd. Vet Rec 133: 89–94. 8212495 10.1136/vr.133.4.89

[2] Peel MM, Palmer GG, Stacpoole AM, Kerr TG (1997) Human lymphadenitis due to *Corynebacterium pseudotuberculosis:* report of ten cases from Australia and review. Clin Infect Dis 24: 185–191. 9114145 10.1093/clinids/24.2.185

[3] Selim SA (2001) Oedematous Skin Disease of Buffalo in Egypt. J Vet Med B Infect Dis Vet Public Health 48: 241–258. 15129580 10.1046/j.1439-0450.2001.00451.x

[4] Pratt SM, Spier SJ, Carroll SP, Vaughan B, Whitcomb MB, et al (2005) Evaluation of clinical characteristics, diagnostic test results, and outcome in horses with internal infection caused by *Corynebacterium pseudotuberculosis*: 30 cases (1995–2003). J Am Vet Med Assoc 227: 441–449. 16121612 10.2460/javma.2005.227.441

[5] Guimarães AS, Carmo FB, Pauletti RB, Seyffert N, Ribeiro D, et al (2011) Caseous Lymphadenitis: Epidemiology, diagnosis, and control. IIOAB J 2: 33–43.

[6] Bregenzer T, Frei R, Ohnacker H, Zimmerli W (1997) *Corynebacterium pseudotuberculosis* infection in a butcher. Clin Microbiol Infect 3: 696–698. 11864217 10.1111/j.1469-0691.1997.tb00482.x

[7] Join-Lambert OF, Ouache M, Canioni D, Beretti JL, Blanche S, et al (2006) *Corynebacterium pseudotuberculosis* necrotizing lymphadenitis in a twelve-year-old patient. Pediatr Infect Dis J 25: 848–851. 16940849 10.1097/01.inf.0000234071.93044.77

[8] Tejedor-Junco MT, Lupiola P, Schulz U, Gutierrez C (2008) Isolation of nitrate-reductase positive *Corynebacterium pseudotuberculosis* from dromedary camels. Trop Anim Health Prod 40: 165–167. 18484117 10.1007/s11250-007-9077-2

[9] Lopes T, Silva A, Thiago R, Carneiro A, Dorella FA, et al (2012) Complete genome sequence of *Corynebacterium pseudotuberculosis* strain Cp267, isolated from a llama. J Bacteriol 194: 3567–3568. 22689248 10.1128/JB.00461-12 PMC3434722

[10] Paton MW, Walker SB, Rose IR, Watt GF (2003) Prevalence of caseous lymphadenitis and usage of caseous lymphadenitis vaccines in sheep flocks. Aust Vet J 81: 91–95. 15084020 10.1111/j.1751-0813.2003.tb11443.x

[11] Guimarães AS, Seyffert N, Bastos BL, Portela RWD, Meyer R, et al (2009) Caseous lymphadenitis in sheep flocks of the state of Minas Gerais, Brazil: prevalence and management surveys. Small Rumin Res 87: 86–91.

[12] Seyffert N, Guimarães AS, Pacheco LGC, Portela RW, Bastos BL, et al (2010) High seroprevalence of caseous lymphadenitis in Brazilian goat herds revealed by *Corynebacterium pseudotuberculosis* secreted proteins-based ELISA. Res Vet Sci 88: 50–55. 19665155 10.1016/j.rvsc.2009.07.002

[13] Guimarães AS, Carmo FB, Heinemann MB, Portela RW, Meyer R, et al (2011) High sero-prevalence of caseous lymphadenitis identified in slaughterhouse samples as a consequence of deficiencies in sheep farm management in the state of Minas Gerais, Brazil. BMC Vet Res 7: 68. 22067701 10.1186/1746-6148-7-68 PMC3256107

[14] Estevao Belchior S, Gallardo A, Abalos A, Jodor N, Jensen O (2006) Actualización sobre lifoadenitis caseosa: El agente etiológico y La enfermedad. Rev Vet Arg 224: 258–278.

[15] Yeruham I, Elad D, Van-Ham M, Shpigel NY, Perl S (1997) *Corynebacterium pseudotuberculosis* infection in Israeli cattle: clinical and epidemiological studies. Vet Rec 140: 423–427. 9149362 10.1136/vr.140.16.423

[16] Yeruham I, Braverman Y, Shpigel NY, Chizov-Ginzburg A, et al (1996) Mastitis in dairy cattle caused by *Corynebacterium pseudotuberculosis* and the feasibility of transmission by houseflies I. Vet Quart. 18: 87–89. 10.1080/01652176.1996.9694623 8903139

[17] Songer JG, Beckenbach K, Marshall MM, Olson GB, Kelley L (1988) Biochemical and genetic characterization of *Corynebacterium pseudotuberculosis* . Am J Vet Res 49: 223–226. 2831763

[18] Sutherland SS, Hart RA, Buller NB (1993) Ribotype analysis of *Corynebacterium pseudotuberculosis* isolates from sheep and goats. Aust Vet J 70: 454–456. 7906938 10.1111/j.1751-0813.1993.tb00851.x

[19] Sutherland SS, Hart RA, Buller NB (1996) Genetic differences between nitrate-negative and nitrate-positive *C. pseudotuberculosis* strains using restriction fragment length polymorphisms. Vet Microbiol 49: 1–9. 8861638 10.1016/0378-1135(95)00146-8

[20] Costa LRR, Spier SJ, Hirsh DC (1998) Comparative molecular characterization of *Corynebacterium pseudotuberculosis* of different origin. Vet Microbiol 62: 135–143. 9695286 10.1016/s0378-1135(98)00202-8

[21] Abreu SRO, Mota RA, Rosinha GMS, Forner O, Pinheiro Júnior JW, et al (2008) Comparação genotípica de isolados de *Corynebacterium pseudotuberculosis* de caprinos e ovinos do sertão de Pernambuco. Pesq Vet Bras 28: 481–187.

[22] Connor KM, Fontaine MC, Rudge K, Baird GJ, Donachie W (2007) Molecular genotyping of multinational ovine and caprine *Corynebacterium pseudotuberculosis* isolates using pulsed-field gel electrophoresis. Vet Res 38: 613–623. 17565908 10.1051/vetres:2007013

[23] Foley JE, Spier SJ, Mihalyi J, Drazenovich N, Leutenegger CM (2004) Molecular epidemiologic features of *Corynebacterium pseudotuberculosis* isolated from horses. Am J Vet Res 65: 1734–1737. 15631043 10.2460/ajvr.2004.65.1734

[24] Guimarães AS, Dorneles EM, Andrade GI, Lage AP, Miyoshi A, et al (2011) Molecular characterization of *Corynebacterium pseudotuberculosis* isolates using ERIC-PCR. Vet Microbiol 153: 299–306. 21733644 10.1016/j.vetmic.2011.06.002

[25] Dorneles EMS, Santana JA, Andrade GI, Santos ELS, Guimarães AS, et al (2012) Molecular characterization of *Corynebacterium pseudotuberculosis* isolated from goats using ERIC-PCR. Gen Mol Res 11: 2051–2059. 10.4238/2012.August.6.9 22911589

[26] Trost E, Ott L, Schneider J, Schröder J, Jaenicke S, et al (2010) The complete genome sequence of *Corynebacterium pseudotuberculosis* FRC41 isolated from a 12-year-old girl with necrotizing lymphadenitis reveals insights into gene-regulatory networks contributing to virulence. BMC Genomics 11: 728. 21192786 10.1186/1471-2164-11-728 PMC3022926

[27] Ruiz JC, D’Afonseca V, Silva A, Ali A, Pinto AC, et al (2011) Evidence for reductive genome evolution and lateral acquisition of virulence functions in two *Corynebacterium pseudotuberculosis* strains. PLoS One 6: e18551. 21533164 10.1371/journal.pone.0018551 PMC3078919

[28] Cerdeira LT, Pinto AC, Schneider MPC, Almeida SS, Santos AR, et al (2011) Whole-Genome Sequence of *Coynebacterium pseudotuberculosis* Strain PAT10 Isolated from Sheep in Patagonia, Argentina. J Bacteriol 193: 6420–6421. 22038974 10.1128/JB.06044-11 PMC3209231

[29] Cerdeira LT, Schneider MPC, Pinto AC, Almeida SS, Santos AR, et al (2011) Complete Genome Sequence of *Corynebacterium pseudotuberculosis* Strain CIP 52.97, isolated from a Horse in Kenya. J Bacteriol 193: 7025–7026. 22123771 10.1128/JB.06293-11 PMC3232848

[30] Silva A, Schneider MPC, Cerdeira L, Barbosa MS, Ramos RTJ, et al (2011) Complete Genome Sequence of *Corynebacterium pseudotuberculosis* I19, a Strain isolated from a Cow in Israel with Bovine Mastitis. J Bacteriol 193: 323–324. 21037006 10.1128/JB.01211-10 PMC3019927

[31] Hassan SS, Schneider MP, Ramos RT, Carneiro AR, Ranieri A, et al (2012) Whole-Genome Sequence of *Corynebacterium pseudotuberculosis* Strain Cp162, Isolated from Camel. J Bacteriol 194: 5718–5719. 23012291 10.1128/JB.01373-12 PMC3458653

[32] Silva A, Ramos RTJ, Carneiro AR, Pinto AC, Soares SC, et al (2012) Complete Genome Sequence of *Corynebacterium pseudotuberculosis* Cp31, Isolated from an Egyptian Buffalo. J Bacteriol 23: 6663–6664. 10.1128/JB.01782-12 PMC3497519 23144408

[33] Pacheco LGG, Pena RR, Castro TLP, Dorella FA, Bahia RC, et al (2007) Multiplex PCR assay for identification of *Corynebacterium pseudotuberculosis* from pure cultures and for rapid detection of this pathogen in clinical samples. J Med Microbiol 56: 1–7. 17374887 10.1099/jmm.0.46997-0

[34] Versalovic J, Koeuth T, Lupskil JR (1991) Distribution of repetitive DNA sequences in eubacteria and application to fingerprinting of bacterial genomes. Nucleic Acids Res 19: 6823–6831. 1762913 10.1093/nar/19.24.6823 PMC329316

[35] Hunter PR, Gaston MA (1988) Numerical index of the discriminatory ability of typing systems: an application of Simpson’s index of diversity. J Clin Microbiol 26: 2465–2466. 3069867 10.1128/jcm.26.11.2465-2466.1988 PMC266921

[36] Struelens MJ (1998) Molecular Epidemiologic Typing Systems of Bacterial Pathogens: Current Issues and Perpectives. Mem Inst Oswaldo Cruz 93: 581–585. 9830521 10.1590/s0074-02761998000500004

[37] Feil EJ, Li BC, Aanensen DM, Hanage WP, Spratt BG (2004) eBURST: Inferring Patterns of Evolutionary Descent among Clusters of Related Bacterial Genotypes from Multilocus Sequence Typing Data. J Bacteriol 186: 1518–1530. 14973027 10.1128/JB.186.5.1518-1530.2004 PMC344416

[38] Salipante SJ, Hall BG (2011) Inadequacies of Minimum Spanning Trees in Molecular Epidemiology. J Clin Microbiol 49: 3568–3575. 21849692 10.1128/JCM.00919-11 PMC3187300

[39] Siegel S (1975) Estatística não-paramétrica. São Paulo: McGraw-Hill.

[40] Matthias G, Lemon J, Fellows I, Singh P (2012) Various Coefficients of Interrater Reliability and Agreemen. R Package Version 0.84. Available: http://CRAN.R-project.org/package=irr. Accessed 2014 May 15.

[41] Struelens M (2002) Molecular typing: a key tool for the surveillance and control of nosocomial infection. Curr Opin Infect Dis 15: 383–385. 12130934 10.1097/00001432-200208000-00005

[42] Li W, Raoult D, Fournier PE (2009) Bacterial strain typing in genomic era. FEMS Microbiol Rev 33: 892–916. 19453749 10.1111/j.1574-6976.2009.00182.x

[43] Foxman B, Zhang L, Koopman JS, Mannig SD, Marrs CF (2005) Choosing an appropriate bacterial typing technique for epidemiologic studies. Epidemiol Perspect Innov 2: 10. 16309556 10.1186/1742-5573-2-10 PMC1308839

[44] Soares SC, Silva A, Trost E, Blom J, Ramos R, et al (2013) The pan-genome of the animal pathogen *Corynebacterium pseudotuberculosis* reveals differences in genome plasticity between the biovar ovis and equi strains. PLoS One 8: e53818. 23342011 10.1371/journal.pone.0053818 PMC3544762

[45] Instituto Brasileiro de Geografia e Estatística (2008) Diretoria de Pesquisas, Coordenação de Agropecuária, Pesquisa da Pecuária Municipal 2007. Censo Agropecuário 2006 [cited 2008]. Available: http://www.ibge.gov.br. Accessed 2014 May 15.

